# New Postage Law

**Published:** 1851-07

**Authors:** 


### OBITUARY.

Died, at his residence, Kenosha, Wis., on the 5th of June, 1851, after a long and painful illness, Doctor David Walker, aged 49 years. The highest testimonials of respect have been paid to his memory by his friends and neighbors.

